# Li-ion Battery Separators, Mechanical Integrity and Failure Mechanisms Leading to Soft and Hard Internal Shorts

**DOI:** 10.1038/srep32578

**Published:** 2016-09-01

**Authors:** Xiaowei Zhang, Elham Sahraei, Kai Wang

**Affiliations:** 1 Impact and Crashworthiness Lab, Massachusetts Institute of Technology, 77 Massachusetts Ave, Room 5-218, Cambridge, MA, 02139, United States; 2 Electric Vehicle Safety Lab, George Mason University, Fairfax, VA, 22030, United States

## Abstract

Separator integrity is an important factor in preventing internal short circuit in lithium-ion batteries. Local penetration tests (nail or conical punch) often produce presumably sporadic results, where in exactly similar cell and test set-ups one cell goes to thermal runaway while the other shows minimal reactions. We conducted an experimental study of the separators under mechanical loading, and discovered two distinct deformation and failure mechanisms, which could explain the difference in short circuit characteristics of otherwise similar tests. Additionally, by investigation of failure modes, we provided a hypothesis about the process of formation of local “soft short circuits” in cells with undetectable failure. Finally, we proposed a criterion for predicting onset of soft short from experimental data.

The risk of mechanical failure and thermal runaway of lithium-ion battery packs in electric vehicles (EVs) subjected to crash loading, imposes severe restrictions on the design of the vehicle and the battery pack. There is a steady progress in testing and modeling of the mechanical properties of lithium-ion battery cells as well as battery components including cathode, anode and separators^1^,^2^,^3^,^4^,^5^,^6^,^7^,^8^,^9^. While there is a lot of information about strength and failure of these batteries, the exact mechanism of short circuit and conditions leading to thermal runaway are still not clearly understood. Local penetration experiments on pouch cells have produced puzzling results: some cells go to short circuit and thermal events under predictable levels of force and displacement while other cells tolerate much larger deformations without any noticeable failure^2^,^10^. The mechanical integrity of battery separator is critical for prevention of internal short circuit. A better understanding of the mechanical behavior and failure mechanisms of the separators may assist in explaining an apparently conflicting response. Also, it can ultimately help to rank the properties of different types of separators under mechanical abuse loading and choose or design the one that would satisfy specific requirements of a battery pack.

Porous membrane separators, such as polyethylene (PE), polypropylene (PP) and polypropylene-polyethylene-polypropylene (PP/PE/PP) made of polyolefin, are widely used in the lithium-ion batteries for EVs^11^,^12^,^13^. They can be manufactured by cold and hot stretch of precursor films with different stretch rates and ratios, followed by annealing until achieving a required porosity^12^,^14^,^15^,^16^,^17^,^18^. The mechanical properties of separators are highly dependent not only on the material that they are made of but also on their manufacturing processes. The so called wet and dry processes of polyolefin bring significant differences. The tensile strength of separators made from wet process in machine direction (MD) and in-plane transverse direction (TD) is comparable, while it is quite different for dry processed separators in these two directions. Researchers have studied effects of using different technologies of producing separators on their properties and suggested characterization procedures^19^,^20^. Also recent publications report on tensile and compressive testing of separators and approaches for modeling them^7^,^21^,^22^,^23^. However, a comprehensive understanding on micro deformations in separator structure and failure mechanisms in more realistic loadings like punch indentation is still missing.

In this study, we tested two typical commercially available dry-processed separators, PE and trilayer (PP/PE/PP), under a variety of loading scenarios. First we documented mechanical strength and failure behavior of the separators under uniaxial tensile and compressive loads. Then we investigated mechanisms of failure under more common biaxial loads. The most important result of the present study is detection and explanation of two distinct failure mechanisms under biaxial loading. We observed a significant difference in the size of short circuit area under these two failure modes. One implication of this finding is in explanation of the extent of electrochemical and thermal reactions when each of these modes is activated. Additionally, the findings explain the so called “soft short” versus “hard short” in the cells subjected to mechanical abuse conditions.

## Results

### Tensile tests

**Figure 1:**
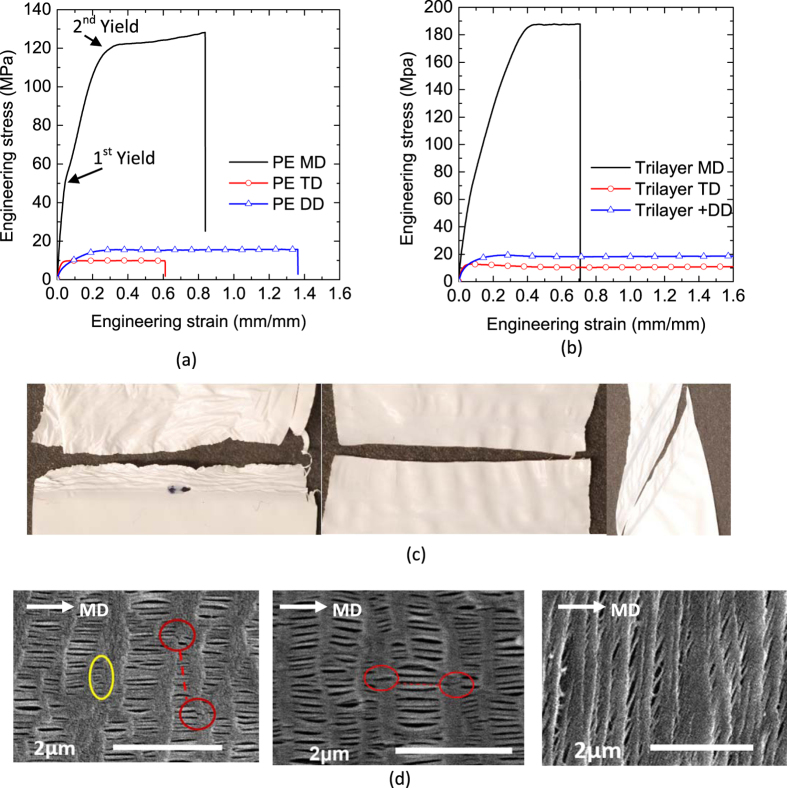
(**a**) Nominal Stress-Strain curve for PE separator, (**b**) Stress-Strain curve for trilayer separator, (**c**) Different failure modes in MD, TD, and DD (from left to right) for PE separator, (**d**) SEM image close to failure region after tensile test along MD, TD, and DD (from left to right) for PE separator.

Figure 1a,b show test results for separators tested in three directions. For dry processed PE and trilayer separators, the strength in diagonal direction (DD) and TD is in the same order, which is much lower than that in MD. This is consistent with the expectation of anisotropic material behavior for dry processed separators. The MD curve has two distinct yield points before failure. Besides the strength levels in each direction, the failure modes are quite different in MD, TD and DD, as shown in Fig. 1c. The failure surfaces in both MD and TD loading were perpendicular to the loading direction, while the fracture surface had a 45° angle in DD loading which made it still perpendicular to TD. The failure surfaces from TD and DD loading were quite smooth. However, the MD loaded specimens had a wrinkled, zigzagged and non-smooth surface. In DD, a significant shear zone was observed and fracture strains were much larger than TD and MD (i.e. DD fracture strain was 1.6 while MD and TD fracture strains were 0.8 and 0.6 for the PE separator). Although the un-deformed separator was solid white, the deformed regions of specimens in TD and DD tensile tests became transparent before failure. We saw similar phenomenon also in punch tests, which will be discussed in the following section. We observed that samples with smaller width had more probability of premature failure due to imperfections in the edge. At the same time, wider specimens provided more repeatable data. Therefore, we recommend for a uniaxial test protocol, to cut the strips at three directions (MD, TD, and DD) with at least 10 mm width to obtain more complete and repeatable information when characterizing a new type of separator.

Figure 1d shows the Scanning Electron Microscope (SEM) pictures of separator sections close to failure zone. The separator structure contains sections of bulk material connected with fibrils (also called crazing zones). During uniaxial tension in MD loading, the bulk area started to become wider and new fibrils (highlighted with yellow oval shape) were created in the bulk material region. In the nominal stress strain curve shown in Fig. 1a, onset of formation of new fibrils corresponds to the second yield point and change of slope. Red circles highlights the cracks in bulk material sections during MD and TD loading. We observed that these cracks in both cases were along MD. In case of MD loading, to have a complete failure surface perpendicular to loading direction, as seen in the tests, the fibrils should break to connect the two cracks in the bulk material. This explains the zigzagged shape of final failure surface, which is formed from alternating cracks in fibrils and bulk regions. However, the mechanism is different in TD loading, where the bulk sections become longer and narrower and the original oval pores between fibrils were enlarged along the minor axis (widening of the openings). The cracks in bulk sections can easily connect to one another through the openings, with minimal fibril failure. Therefore the surface of failed region contains smooth fibrils, and can propagate much faster. In DD loading, the localized shear zone shows large rotation of fibrils, which is closing the holes in the crazed region. The bulk material regions are getting stretched and becoming narrower as well, which was similar to deformation of bulk sections in TD loading.

### Through thickness compression tests

**Figure 2:**
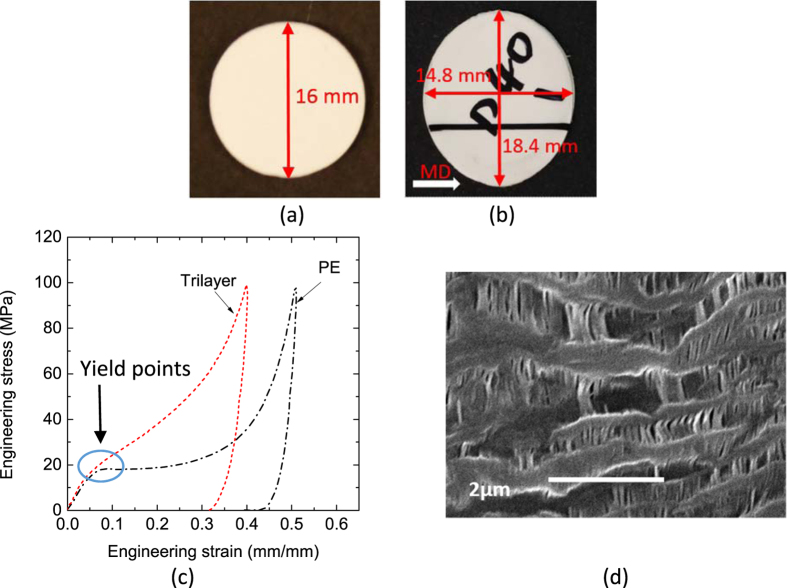
(**a**) Specimen before out-of-plane compression test, (**b**) Specimen after test, (**c**) Engineering stress strain curves, and (**d**) SEM image after the test.

Due to a thin-film nature and operating environment, the separator must sustain a large out-of-plane compression load from electrodes during charge and discharge or abuse loading. It has been reported that the engineering-stress and strain curves are different in thickness direction compared to those in MD, TD and DD^24^. We tested the separators under compression by stacking separators together with initial compression stress of 0.5 MPa. We found that with stacking more layers of the separator, a better repeatability can be achieved. Therefore, 40-layer stacked separator specimens were used. Five repeatable tests were conducted for each separator. The dimensions of PE separator before and after tests are shown in Fig. 2a,b and the nominal stress-strain curves are presented in Fig. 2c. Compression tests also reveal the anisotropic nature of the dry processed separators by the oval shape of specimens after the test. Under through thickness compression, the material in TD, which has lower strength, got stretched while it shrunk in MD. The separators experienced obvious yield points under the applied compression. After the yield point, the stress was growing exponentially until almost full densification. An SEM image of this separator (Fig. 2d) shows that bulk material regions have been stretched along width and length, which in turned caused local buckling of fibrils. This explains the lengthening in TD and shrinking in MD seen in Fig. 2b.

### Biaxial punch tests and two failure modes

**Figure 3:**
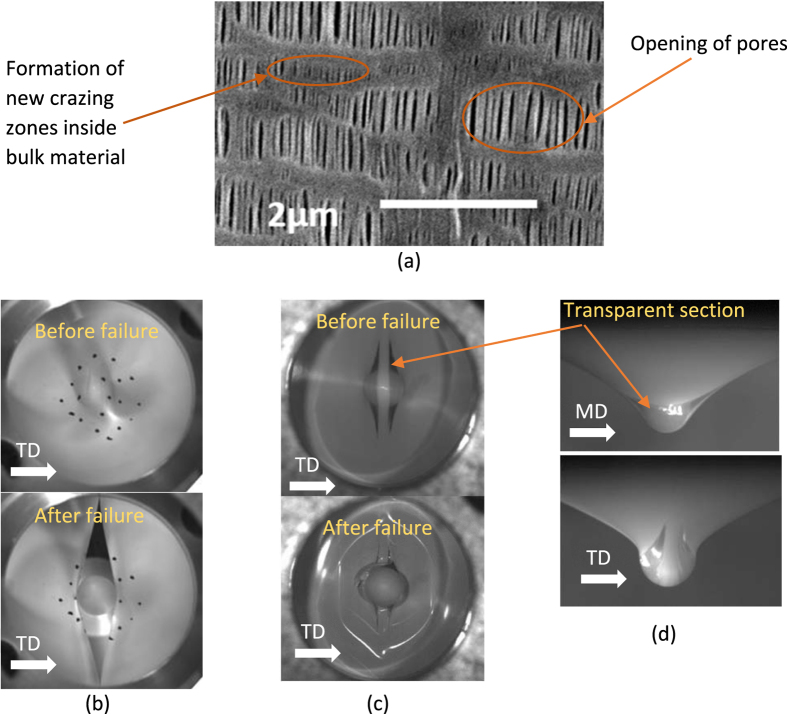
(**a**) SEM image of PE separator, (**b**) Mode A failure, (**c**) Mode B failure, and (**d**) Additional views of transparent area before failure in Mode B.

A more representative loading case for separators is a punch intrusion. The separator may go under this kind of loading in most real world mechanical abuse scenarios. It represents combined in-plane biaxial tension and out-of-plane compression. A punch test with a small radius punch head is one of the standard abuse tests for lithium-ion battery separators. It is performed with a punch of 3.2 mm in diameter according to ASTM F1306-90, and usually referred to as a puncture test^25^. Punch tests can eliminate the errors induced by imperfections on the edge due to cutting method, and thus provide a good estimate of material properties and failure strains. Punch tests were carried out with a punch head diameter of 25.4 mm, 12.7 mm, 6.4 mm and 3.2 mm. The SEM image (Fig. 3a) shows that separator under punch loading gets both effects of TD and MD tensile loads. Formation of new fibrils in the bulk material regions is an effect similar to MD uniaxial loads, while opening of the pores is one seen in TD loading.

An interesting observation is that two different failure modes co-exist for dry processed separators, as shown in Fig. 3b,c. In the first and more common one (named failure Mode A, seen in Fig. 3b), the cracks form along MD, featuring a large diagonal slit. The smooth surface of the tear along MD for this failure mode is similar to what was observed in uniaxial TD loading cases in Fig. 1c (middle). The other failure mode creates a zig-zag surface along TD (named failure Mode B, seen in Fig. 3c), similar to what was observed in uniaxial MD loading cases in Fig. 1c (left). Failure Mode A happens under a smaller punch force and a lower punch displacement. The sample would reach failure Mode B only if it passes force and displacement levels associated with Mode A without failure. However, a transparent section is formed along MD when the forces and displacements exceeds values corresponding to Mode A, see Fig. 3d. The difference in curvatures of deformed shape from side views along MD and TD clearly show effect of high anisotropy.

**Figure 4:**
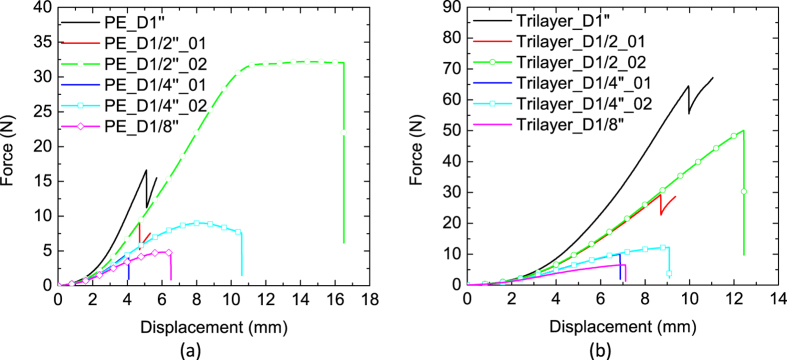
Force-displacement curves from punch loading with different punch sizes for (**a**) PE separator and (**b**) Trilayer separator.

Figure 4a,b shows the force-displacement curves of each separator with different sizes of punch head. Specimens failed in Mode A are shown with solid lines and the ones failed in Mode B are shown with circular symbols. In each punch size, a failure in Mode A is associated with much smaller force and displacement, when compared to a Mode B failure. While the tests were repeated five times, to keep the figure clear, only one representative sample curve from each failure mode case is plotted. For the PE separator, with the punch diameter of 1 inch (25 mm), all the five specimens failed in Mode A. When the punch head size decreased to 1/2 inch (12.5 mm) diameter, three out of five specimens failed in Mode A, and two of them failed in Mode B. With punch head diameter of 1/4 inch (6.4 mm), only one specimen in five repeated tests failed in Mode A and the rest failed in Mode B. With punch diameter of 1/8 inch (3.2 mm), all the five specimens failed in Mode B. We observed similar trends for the trilayer separator.

A relatively free flow of the material that is in contact with the punch head is needed to allow deformation beyond Mode A, without localization in one point of the stretched transparent section, until reaching Mode B. This was accommodated in the above tests due to use of Teflon punch heads which eliminated friction. For large punch heads, it is not easy to allow all the material under the punch head to flow freely and deform relatively “uniformly”. Therefore failure Mode B is mostly observed only in smaller punch sizes.

### Discussion on formation of distinct short circuits

#### Hypotesis, Soft verus Hard Short Circuit

In punch test of the whole battery cells, due to the friction from cathode and anode particles, the separator is mostly expected to fail in Mode A, and not to reach Mode B. With the start of thinning and appearance of transparent section, friction would lead to instant localization. However, failure in Mode B could be observed in extremely small punch sizes (pin or needle tests). It is important to understand whether a cell that has not failed in Mode A is still safe, while going through thinnening and formation of transparent area that is associated with deformations beyond Mode A. We measured the electrical resistance of the separator by soaking it in DMC and locating it between two electrodes, and repeated the measurement on a separator that had gone to thinnening and transparency. Results showed a drop of 70% (from 782 k ohm to 223 k ohm) in the resistance. This low resistance area in a cell would cause a nonuniform distribution of electrochemical reactions and electric current inside the cell.

**Figure 5:**
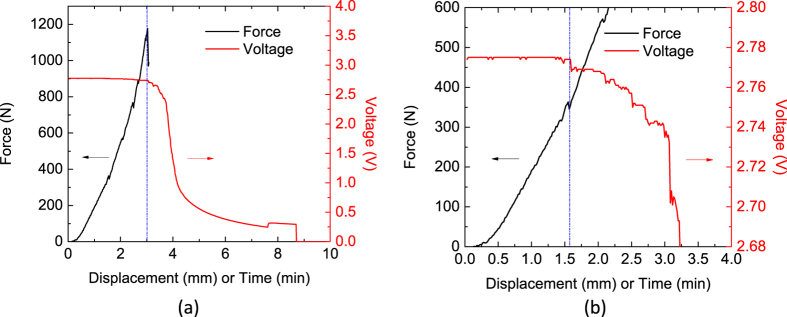
(**a**) Original global curve shows a hard short observed at 3 mm displacement, (**b**) Zommed curve, shows a soft short at 1.5 mm displacemet.

In a full cell experiment previously reported by Sahraei *et al*.^6^, the authors performed punch tests with various punch sizes on different pouch cells, until detecting a short circuit from drop in punch force and cell voltage. While peak force and displacements to failure usually followed a linear trend with punch size, they observed that in a conical punch (smallest punch) test of a large pouch cell, the peak values were much beyond what was expected for that punch size. With the new understanding about two failure modes discussed above, we have a hypothesis that this cell had a separator failure in Mode B, while all other cells had failed in mode A. As shown in Fig. 5a, in this test, short circuit was detected at a force of 1200N and punch displacement of 3 mm. Scrutinizing in the cell voltage with a higher precision showed that a small decrease in voltage had started around the force and displacement values which corresponded to failure in other cells. The authors concluded that a soft short had started at that point. The soft short was not detected as a failure, and loading continued until much larger force levels. The above cell was not opened after initiation of soft short, so we do not have a strict proof on mode of failure of separator, also we could not repeat the test, because such cases happen in statistically small number of cases which makes design of proper experiment cost prohibitive. However, the above hypothesis has important implications which makes it suitable topic for future investigations. It means that failure Mode A is of an utmost importance: it can trigger soft shorts due to thinning and probable opening of the pores in the transparent area, without causing a full detectable failure in the cell.

Please note, we are describing the soft and hard short circuits that are due to mechanical loading. We acknowledge that soft short can be developed due to growth of dendrite or other local defects in the cell production, and in those cases, the failure mechanism might be different.

#### Extreme versus mild thermal events

**Figure 6:**
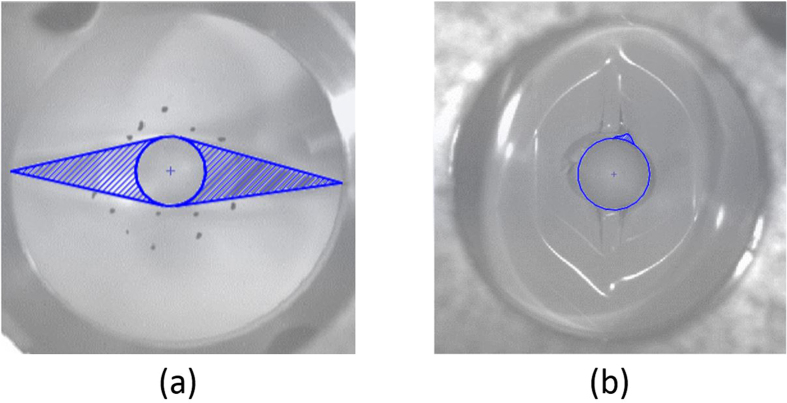
Short circuit area due to Mode A failure (**a**), versus the area in a Mode B Failure (**b**).

The two failure modes may also provide some explanation about lack of repeatability in nail penetration tests, when in some tests cell may fail in Mode A and in some it could fail in Mode B. In Fig. 6, we have highlighted the geometry of possible contact between materials on the two sides of a separator when it fails in Mode A or Mode B. The much larger contact area due to a Mode A failure may result in a much more violent thermal event in this case when compared to the small area seen in Mode B failure.

#### Separator Failure Criterion

**Figure 7:**
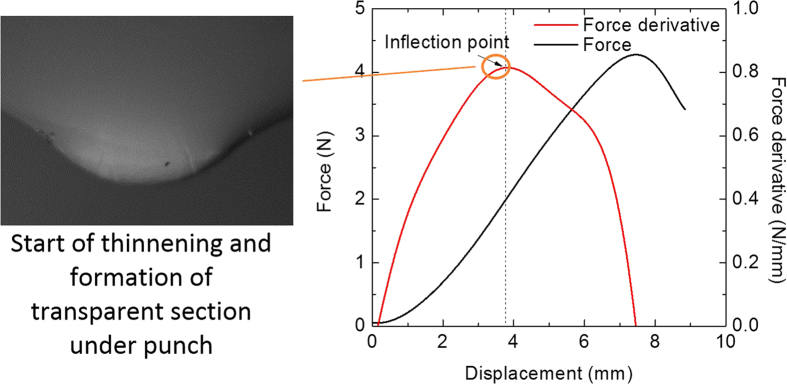
Onset of thinning and formation of transparent area in the PE separator.

For the design purpose, where a proper measure should be chosen for optimum protection against deformation, it is important that the displacement leading to onset of Mode A is used as failure criterion. Otherwise the cell can undergo a soft short without detection. While some punch tests can lead to failure in Mode A, and therefore determining the load-displacements associated with Mode A would be directly possible, in the cases of tests leading to Mode B failure, it is harder to estimate what the values for Mode A failure would be. To develop a failure criterion, the onset of thinning and formation of transparent area under the punch should be detected. We looked very closely into this by reviewing magnified images of the contact area under the punch in order to detect this point (see Fig. 7). We discovered that the onset of thinning observed from the tests could be detected on the load displacement curve from a change in convexity, i.e. by the inflection point. The peak value in the slope of force-displacement curve, as shown in Fig. 7 with a blue curve, shows the point of inflection which is where a thin and transparent area in the separator starts to appear. According to these findings, the critical displacement corresponding to the onset of failure point A can be calculated from:


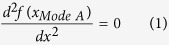


where *f* and *x* denote respectively the punch force, and displacement, and *x*_*Mode A*_ is the punch displacement at the onset of Mode A failure.

#### Other types of Separators

It should be noted that while dry-processed separators are commonly used in EV battery applications due to their low manufacturing cost, recent advantages of wet-processed ceramic coated separators have made them a viable choice. Authors have studied failure mechanisms of those separators as well as non-woven separators in a concurrent publication^26^, and it should be acknowledged that those separators have completely different failure mechanisms.

## Conclusions

We performed a comprehensive experimental study to characterize mechanical properties of two types of separators: dry processed PE and trilayer. We characterized these separators under uniaxial tensile loading along machine direction, transverse direction, and diagonal direction. Additionally, we documented results on through thickness compression tests and biaxial tensile tests. Dry processed separators, currently widely used in commercial batteries, have two different failure modes. Understanding of these failure modes and their characteristics gives insight about inconsistent nail penetration results, where a failure in Mode A can result in an extreme thermal episode, but a repeat of the test leading to failure Mode B can be much less eventful. Additionally, a criterion for displacements that a separator can tolerate without having a soft failure was proposed. It could be used to design cell manufacturing procedures or battery protective structures while keeping in mind level of tolerance of the separator associated with a soft short.

## Methods

The separators were purchased from MTI Corp with specifications given in Table 1. Uniaxial tensile tests were conducted with five different width of the strip specimens: 5 mm, 10 mm, 15 mm, 20 mm and 25 mm. The specimens were cut with sharp razor (no visible tear along cutting edges) along MD, TD and DD with five repeats in each direction.

According to ASTM D882 for thin film, the separator should be cut with a uniform width in the range from 5 mm to 25.4 mm. With limited height from original separator roll, the total length of the strip specimen was fixed at 60 mm and gauge length as 35 mm. For polymeric separators, which usually do not have visible local necking before failure in uniaxial tensile tests, fracture determines the range of the material’s elasticity and plasticity curve. In order to eliminate the edge damage, the separator was sandwiched between 5-mm Cartesian graph paper for cutting. The strip specimens were tested on an Instron 5944 uniaxial tensile machine with 100 N load cell and the nominal strain was obtained from digital image correlation (DIC) method (Vic 2D, 2009). The loading speed was 25 mm/min.

For the compression tests, samples were cut by 16 mm diameter hammer-driven hole punch at one time from folded separator sheets to ensure that all the layers had the same orientation. The stacked specimens were tested in a MTS uniaxial test machine with 200 kN load cell at a strain rate of 0.012/s in order to have the same strain rate as in the uniaxial tensile tests.

**Figure 8:**
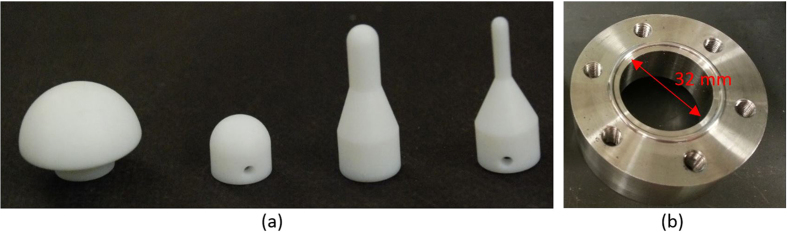
(**a**) Teflon punch head with diameter of 25.4 mm, 12.7 mm, 6.4 mm and 3.2 mm (from left to right); (**b**) biaxial loading fixture.

Biaxial punch tests were performed with punch sizes of 1/8 to 1 inch. The punch heads were machined from teflon bulk to minimize the contact friction. The specimens were cut by a hammer driven hole punch with a diameter of 45 mm. The sample test diameter (see Figure A1 b) was 32 mm. Each type of separator was tested with four different punch heads. Each test scenario was repeated five times. The loading speed was chosen as 12 mm/min to ensure a comparable strain rate to the uniaxial tensile tests at onset of fracture. The fixture is shown in Fig. 8.

## Additional Information

**How to cite this article**: Zhang, X. *et al*. Li-ion Battery Separators, Mechanical Integrity and Failure Mechanisms Leading to Soft and Hard Internal Shorts. *Sci. Rep.* **6**, 32578; doi: 10.1038/srep32578 (2016).

## Figures and Tables

**Table 1:** Separators’ specifications.

	1	2
Material	PE	PP/PE/PP
Process	Dry	Dry
Thickness (*μm*)	25	25
Porosity	36–46%	39%
Roll Width	85 mm	85 mm
